# Unbiased Taxonomic Annotation of Metagenomic Samples

**DOI:** 10.1089/cmb.2017.0144

**Published:** 2018-03-01

**Authors:** Bruno Fosso, Graziano Pesole, Francesc Rosselló, Gabriel Valiente

**Affiliations:** ^1^ Institute of Biomembranes and Bioenergetics, Consiglio Nazionale delle Ricerche, Bari, Italy.; ^2^ Department of Mathematics and Computer Science, Balearic Islands Health Research Institute (IdISBa), University of the Balearic Islands, Palma de Mallorca, Spain.; ^3^ Algorithms, Bioinformatics, Complexity and Formal Methods Research Group, Technical University of Catalonia, Barcelona, Spain.

**Keywords:** classification, correlation, metagenomics, set cover, taxonomic annotation

## Abstract

**The classification of reads from a metagenomic sample using a reference taxonomy is usually based on first mapping the reads to the reference sequences and then classifying each read at a node under the lowest common ancestor of the candidate sequences in the reference taxonomy with the least classification error. However, this taxonomic annotation can be biased by an imbalanced taxonomy and also by the presence of multiple nodes in the taxonomy with the least classification error for a given read. In this article, we show that the Rand index is a better indicator of classification error than the often used area under the** **receiver operating characteristic (ROC) curve and** ***F*-measure for both balanced and imbalanced reference taxonomies, and we also address the second source of bias by reducing the taxonomic annotation problem for a whole metagenomic sample to a set cover problem, for which a logarithmic approximation can be obtained in linear time and an exact solution can be obtained by integer linear programming. Experimental results with a proof-of-concept implementation of the set cover approach to taxonomic annotation in a next release of the TANGO software show that the set cover approach further reduces ambiguity in the taxonomic annotation obtained with TANGO without distorting the relative abundance profile of the metagenomic sample.**

## 1. Introduction

Next-generation sequencing technologies have moved forward the development of metagenomics, a new field of science devoted to the study of microbial communities by the analysis of their genomic content, directly sequenced from the environment (Kunin et al., 2008; Wooley et al., 2010; Thomas et al., 2012). A sequenced metagenomic sample consists of a large number of relatively short DNA or RNA fragments, called reads, and one of the first steps in the computational analysis of a metagenomic sample is the identification of the organisms present in the sequenced environment and their relative abundance, that is, the classification of the metagenomic sample.

In this article, we focus on the taxonomic annotation problem, that is, the classification of the reads from a metagenomic sample using a reference taxonomy, for which we adapt some basic notions from statistical classification in machine learning. We abstract away from the computational problem of mapping reads to reference sequences, and assume that a set of candidate sequences in a reference taxonomy is given for each read in the metagenomic sample to be classified. These candidate sequences are usually obtained either by sequence composition methods (those reference sequences with oligonucleotide frequencies within a given distance threshold to the oligonucleotide frequencies of the read) or by sequence similarity methods (those reference sequences that the read can be aligned to within a given threshold of sequence similarity, or those reference sequences that the read can be mapped to with at most a given number of mismatches).

In a statistical binary classification problem, the confusion matrix (Table 1) shows the number of correctly and incorrectly classified instances of each class. True positives (*TP*) are the correctly classified positive instances, true negatives (*TN*) are the correctly classified negative instances, false positives (*FP*) are the misclassified negative instances, and false negatives (*FN*) are the misclassified positive instances. The *TP* rate, sensitivity, or recall *R* of a classification is the ratio $TPR = TP / ( TP + FN )$ of *TP* to the total number of positive instances, the *FP* rate is the ratio $FPR = FP / ( FP + TN )$ of *FP* to the total number of negative instances, the *TN* rate or specificity is the ratio $TNR = TN / ( FP + TN )$ of *TN* to the total number of negative instances, and the *FN* rate is the ratio $FNR = FN / ( TP + FN )$ of *FN* to the total number of positive instances. Furthermore, the precision of a classification is the ratio $P = TP / ( TP + FP )$ of *TP* to the total number of positive predictions. They are usually combined into a single indicator of classification error as either the area under the receiver operating characteristic (ROC) curve $AUC = ( TPR - FPR + 1 ) / 2$ or the *F*-measure, which is the harmonic mean $F = 2 / ( 1 / P + 1 / R )$ of precision and recall (Powers, 2011).

**Table 1.** Confusion Matrix for a Binary Classification Problem

	*Positive prediction*	*Negative prediction*
Positive class	*TP*	*FN*
Negative class	*FP*	*TN*

*FN*, false negative; *FP*, false positive; *TN*, true negative; *TP*, true positive.

**FIG. 1.**
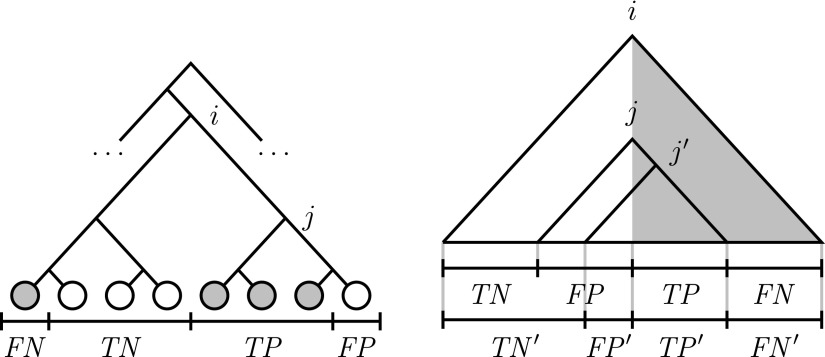
Classifying a read using a reference taxonomy. The grayed leaves are the candidate sequences for the classification of the read, and node *i* is their lowest common ancestor in the reference taxonomy. The taxonomic annotation of the read at node *i* implies the absence of *TN* and *FN*. With a taxonomic annotation of the read at node *j*, however, the grayed leaves under node *j* are the true positives, the remaining grayed leaves are the *FN*, the remaining leaves under node *j* are the false positives, and the still remaining leaves under node *i* are the *TN* of the metagenomic classification problem. *FN*, false negatives; FP, false positives; *TN*, true negatives; *TP*, true positives

In a metagenomic classification problem, the annotation of a read as coming from a particular sequence in a reference taxonomy often involves solving the ambiguity of multiple candidate sequences, caused among other factors by reads being not long enough to ensure a unique identification of the reference sequences they come from. Reference taxonomies are rooted trees, with the leaves labeled by sequences at the taxonomic rank of species or strain, and these ambiguities are solved by annotating reads as coming from internal nodes, at higher taxonomic ranks in the reference taxonomy. When classifying a read as coming from an internal node in a reference taxonomy (Fig. 1), the leaves under the internal node are *TP* if they are labeled by candidate sequences, otherwise they are *FP*, and the remaining leaves under the lowest common ancestor (LCA) of the candidate sequences are *FN* if they are labeled by candidate sequences; otherwise, they are *TN*. Annotating a read as coming from the LCA of the candidate sequences in a reference taxonomy (Huson and Weber, 2013) maximizes precision, as in that case there are no *TN* and no *FN*, but at the expense of specificity, because the number of *FP* in a reference taxonomy can be very large. Annotating a read as coming from an internal node with the largest *F*-measure value (Clemente et al., 2011; Alonso et al., 2013; Fosso et al., 2015, 2017) minimizes the classification error as a combination of precision and sensitivity.

However, there are at least two sources of bias in the taxonomic annotation of a metagenomic sample. One the one hand, reference taxonomies are imbalanced, that is, the instances of one class significantly outnumber the instances of the other classes, and this can be observed at any taxonomic rank. For example, the NCBI Taxonomy (Federhen, 2012, 2015), which is the most comprehensive taxonomic reference to date, includes as of March 13, 2017, an imbalanced number of sequences for Bacteria (1,412,065), Eukaryota (685,380), and Archaea (27,322). Within the Bacteria, for example, there is also an imbalanced number of sequences for the Actinobacteria (593,837), Proteobacteria (440,315), Firmicutes (245,632), Bacteroidetes (77,866), Planctomycetes (8899), Fusobacteria (7789), and others (37,727).

In a statistical binary classification problem, imbalanced data sets result in a good coverage of the positive instances and a frequent misclassification of the negative instances, since most of the standard machine learning algorithms consider a balanced training set (López et al., 2013). In a metagenomic classification problem, an imbalanced reference taxonomy may also yield an imbalance between the positive and negative classes, because the larger the clade of the LCA in a reference taxonomy of the candidate sequences for a read, the larger the negative class for the classification of the read. In this article, we show that this is in general not the case, and we also show that the Rand index is a better indicator of classification error than the often used area under the ROC curve and *F*-measure, when the reference taxonomy is imbalanced and also for balanced reference taxonomies.

Another source of bias in the taxonomic annotation of a metagenomic sample lies in the existence of multiple candidate nodes in a reference taxonomy with the least classification error for a given read, one of which is usually chosen arbitrarily for the taxonomic annotation of the read (Clemente et al., 2011; Alonso et al., 2013). Instead of breaking ties independently for each read in a metagenomic sample, we show in this article that the shift from a one-sequence-read-at-a-time view to a whole-set-of-sequence-reads view yields a better resolution of any remaining ambiguities in the taxonomic annotation of a metagenomic sample.

## 2. Taxonomic Annotation Using Imbalanced Reference Taxonomies

Recall that in a metagenomic classification problem, an imbalanced reference taxonomy yields an imbalance between the positive and negative classes. Let us define the balance ratio of a classification problem as the ratio of the size of the positive class to the size of the negative class.

**Definition 1.** *Let TP, TN, FP, and FN be the number of TP, FP, TN, and FN in a binary classification problem. The balance ratio of the classification problem is* $( TP + FN ) / ( FP + TN )$.

Recall also that the reference taxonomies used in metagenomic classification are highly imbalanced. It turns out that balanced and imbalanced reference taxonomies yield exactly the same metagenomic classification problems, as long as they have the same number of internal nodes. Some evidence supporting this observation follows.

The topology of the most possible balanced binary reference taxonomy is a complete binary tree, as every internal node (and also the root) has two descendant clades of exactly the same size. On the other hand, the topology of the least possible balanced binary reference taxonomy is a rooted caterpillar, as every internal node (and also the root) has one big descendant clade and one small (with only one node) descendant clade. Borrowing the notion of total cophenetic index for phylogenetic trees (Mir et al., 2013) to measure the balance of reference taxonomies, complete binary trees have indeed the minimum value while rooted caterpillars have the maximum value. Notice, however, that the total cophenetic index of the NCBI Taxonomy (Federhen, 2012, 2015) restricted to the standard taxonomic ranks (Kingdom, Phylum, Class, Order, Family, Genus, and Species) is 206,110,330,551, which represents only 0.00032060% of the interval between the minimum value (727,931) and the maximum value (64,288,827,123,576,010) for the number of taxa in the restricted NCBI Taxonomy.

Now, in a metagenomic classification problem, any subset of the leaves of a reference taxonomy may be labeled by the candidate sequences for the classification of a given read. For a given subset of the leaves of a reference taxonomy, each candidate internal node (at or under the LCA of the subset of the leaves) for the taxonomic annotation of the read yields a certain number of *TP*, *FP*, *TN*, and *FN*. For example, for the reference taxonomy in Figure 1, the subset of grayed leaves yields, for the candidate internal node *j*, a metagenomic classification problem with $TP = 3$, $FP = 1$, $TN = 3$, $FN = 1$, and thus, balance ratio $( 3 + 1 ) / ( 1 + 3 ) = 1$. Table 2 shows the distribution of the number of *TP*, *FP*, *TN*, and *FN* for all subsets of the leaves of a reference taxonomy and for every candidate internal node for the taxonomic annotation of a read having as candidate sequences the subset of the leaves, for both a complete binary tree and a rooted caterpillar with 8 leaves.

**Table 2.** Distribution of *TP*, *FP*, *TN*, *FN* Values (Left) and Distribution of $TP + FN$ Values (Right) in Metagenomic Classification Problems for Different Taxonomic Reference Topologies: Complete Binary Tree (B) and Rooted Caterpillar (C) with Eight Leaves

${ \rm{TP}}$	${ \rm{FP}}$	${ \rm{TN}}$	${ \rm{FN}}$	*B*	*C*	${ \rm{TP + FN}}$	*Count*
0	2	0	6	4	1	1	56
0	2	1	5	24	6	2	196
0	2	2	4	60	15	3	392
0	2	3	3	80	20	4	490
$\cdots$	$\cdots$	$\cdots$	$\cdots$	$\cdots$	$\cdots$	5	392
7	0	1	0	0	1	6	196
7	1	0	0	8	8	7	56
8	0	0	0	1	1	8	7

The resulting distribution of $TP + FN$ values (Table 2, right) is exactly the same in both cases, and thus, a complete binary tree and a rooted caterpillar with the same number of leaves have the same balance ratio. In fact, any two reference taxonomies for the same taxa have the same balance ratio as long as they have the same number of internal nodes, because they yield a metagenomic classification problem for any subset of the leaves and for any candidate internal node, and $TP + FN$ equals the number of leaves in the subset.

Let us assume that the reads in a metagenomic sample to be classified come from known sequences in a reference taxonomy, as it is usually the case in the taxonomic annotation of metagenomic samples, whereas reads coming from novel sequences are annotated by using clustering methods instead. Given a read and a set of candidate sequences in a reference taxonomy, the taxonomic annotation of the read at a certain node in the clade of the LCA in the reference taxonomy of the set of candidate sequences can then be taken to be correct if, and only if, the candidate sequence that the read comes from lies in the clade of the node at which it is annotated.

Based on this observation, we have studied the performance of some of the most often used indicators of classification error: the Yule $\phi$ (Yule, 1912), also known as Matthews correlation coefficient (Matthews, 1975), the area under the ROC curve, the Youden *J* (Youden, 1950), the *F*-measure (Powers, 2011), the Jaccard similarity coefficient (Jaccard, 1901), and the Rand index (Rand, 1971), in the taxonomic annotation of metagenomic samples.

**Definition 2.** *Let TP, TN, FP, and FN be the number of TP, FP, TN, and FN in a binary classification problem.*

• *The Yule* $\phi$ *is given by*
  \begin{align*}
\phi = { \frac { TP \;TN - FP \;FN }  { \sqrt { ( TP + FP ) ( TP + FN ) ( TN + FP ) ( TN + FN ) } } } 
\end{align*}
• *The Youden J is given by*
  \begin{align*}
J = { \frac { TP \,TN - FP \,FN }  { ( TP + FN ) ( FP + TN ) } } 
\end{align*}
• *The area under the ROC curve is given by*
  \begin{align*}
AUC = \frac { 1 }  { 2 } \left( { { \frac { TP }  { TP + FN } } + { \frac { TN }  { FP + TN } } } \right)
\end{align*}
• *The F-measure is given by*
  \begin{align*}
F = { \frac { 2 \;TP }  { 2 \;TP + FP + FN } } 
\end{align*}
• *The Jaccard similarity coefficient is given by*
  \begin{align*}
C = { \frac { TP }  { TP + FP + FN } } 
\end{align*}
• *The Rand index is given by*
  \begin{align*}
R = { \frac { TP + TN }  { TP + FP + TN + FN } } 
\end{align*}

If the denominator in any of these formulas is zero, the value of the indicator is arbitrarily set to zero.

We have computed the value of all these indicators of classification error for each possible set of candidate sequences in a reference taxonomy and for each possible candidate node for the taxonomic annotation of a read coming from each of the candidate sequences, for different taxonomic reference topologies: complete binary trees that have the largest possible balance but yield the least balanced metagenomic classification problems, and rooted caterpillars that have the smallest possible balance but yield the most balanced metagenomic classification problems. For these classification problems, we have counted the number of times the taxonomic annotation is correct, that is, the number of times the candidate sequence that the read comes from lies in the clade of the node in the reference taxonomy at which it is annotated.

The results (Table 3) show that the worst indicator of classification error is the Yule $\phi$, followed by *AUC* and the Youden *J* (which are equivalent, as $J = 2{ \kern 1pt} AUC - 1$), the *F*-measure and the Jaccard similarity coefficient *C* (which are also equivalent, as $C = F / ( 2 - F )$), and that the Rand index *R* is the best indicator of classification error for the taxonomic annotation of metagenomic samples. This can be explained by the fact that in a metagenomic classification problem, we focus on the correct classification of a correct taxonomic annotation while in a statistical classification problem in machine learning, where both positive and negative instances are taken into account, correlation measures such as the Yule $\phi$ (which is equivalent to the Pearson correlation coefficient for binary classification problems) often are the best indicators of classification error.

**Table 3.** Total Number of Correct Taxonomic Annotations Under the Yule ($\phi$), the Area Under the Receiver Operating Characteristic (ROC) Curve (*A*) or the Youden *J*, the *F*-Measure (*F*) or the Jaccard Similarity Coefficient, and the Rand Index (*R*) for Reads Coming from Known Sequences, for Different Taxonomic Reference Topologies (Complete Binary Tree and Rooted Caterpillar) with *n* Leaves

*Complete binary tree*															
*n*	2	3	4	5	6	7	8	9	10	11	12	13	14	15	16
$\phi$	4	14	40	70	262	306	824	1450	4318	6156	17,064	28,158	63,378	118,292	270,448
*A*	4	14	40	70	262	306	920	1530	4726	6316	22,056	29,528	79,322	138,477	352,496
*F*	4	12	32	78	220	407	984	2234	5188	10,251	24,844	49,019	112,812	235,322	493,856
*R*	4	12	48	90	344	485	1544	2742	8308	11,845	37,764	54,757	154,012	239,147	672,416

*Rooted caterpillar*															
*n*	2	3	4	5	6	7	8	9	10	11	12	13	14	15	16
$\phi$	4	14	38	80	203	388	945	1961	4344	8592	20,152	39,474	88,063	183,603	398,700
*A*	4	14	38	80	211	384	973	1952	4628	8346	22,230	38,088	94,962	188,986	421,697
*F*	4	12	32	79	195	441	1024	2270	5104	10,994	24,491	51,959	113,305	241,277	518,937
*R*	4	12	36	89	222	512	1191	2652	5949	12,971	28,459	61,189	132,263	281,547	602,076

Now, the taxonomic annotation of a metagenomic sample involves obtaining the candidate nodes in a reference taxonomy with the least classification error (for a given indicator) for each of the reads in the metagenomic sample. We have proved in Clemente et al. (2011) that, when the *F*-measure is taken as indicator, it suffices to consider candidate nodes that are either candidate sequences themselves, or the LCA of two or more candidate sequences in the reference taxonomy. That is, it suffices to consider as candidate nodes the LCA skeleton tree (Fischer and Huson, 2010) of the set of candidate sequences for a given read.

We prove below that it also suffices to consider the LCA skeleton tree when the Yule $\phi$, the Youden *J*, the area under the ROC curve, the Jaccard similarity coefficient, or the Rand index is taken as indicator of classification error.

Let *T* be a reference taxonomy, let ${{M}_i}$ be the set of candidate sequences for the classification of read *i*, and let ${{T}_i}$ be the subtree of *T* rooted at the LCA of ${{M}_i}$. See Figure 1 for a schematic view.

**Definition 3.** *A node j in T_i_ is called relevant if it is equal to a candidate sequence in M_i_ or equal to the LCA of two or more candidate sequences in M_i_*.

Also, for every node *j* in *T_i_*, let ${T_{i , j}}$ be the subtree of *T_i_* rooted at *j*, let *L_i_* be the set of all candidate sequences in *T_i_*, and let *N_i_* be the set of all candidate sequences in *T_i_* that do not belong to *M_i_* (hence, ${L_i} = {M_i} \cup {N_i}$). Similarly, let ${M_{i , j}}$ be the set of all candidate sequences in ${T_{i , j}}$ that belong to *M_i_*, let ${N_{i , j}}$ be the set of all candidate sequences in ${T_{i , j}}$ that do not belong to ${M_{i , j}}$, and let ${L_{i , j}} = {M_{i , j}} \cup {N_{i , j}}$. Using this notation, for the taxonomic annotation at node *j* of a read *i* with candidate sequences *M_i_* (Fig. 1), the *TP* are $T{P_{i , j}}$ = ${M_{i , j}}$, the *FP* are $F{P_{i , j}}$ = ${N_{i , j}}$, the *TN* are $T{N_{i , j}}$ = ${N_i} \backslash {N_{i , j}}$, and the *FN* are $F{N_{i , j}}$ = ${M_i} \backslash {M_{i , j}}$. Let ${C_{i , j}}$ be the Jaccard correlation coefficient for node *j* in *T_i_*, that is, ${C_{i , j}} = T{P_{i , j}} / ( T{P_{i , j}} + F{P_{i , j}} + F{N_{i , j}} )$. Similarly, let ${Y_{i , j}}$, ${J_{i , j}}$, ${A_{i , j}}$, and ${R_{i , j}}$ be the Yule $\phi$, the Youden *J*, the area under the ROC curve, and the Rand index for node *j* in *T_i_*, respectively. We have:

**Theorem 1.** *For each node j in T_i_, there exists a relevant node* $j ^{\prime}$ *such that* ${Y_{i , j ^{\prime} }} \; \ge \;{Y_{i , j}}$*,* ${J_{i , j ^{\prime} }} \; \ge \;{J_{i , j}}$*,* ${A_{i , j ^{\prime} }} \; \ge \;{A_{i , j}}$*,*${C_{i , j ^{\prime} }} \; \ge \;{C_{i , j}}$*, and* ${R_{i , j ^{\prime} }} \; \ge \;{R_{i , j}}$.

*Proof.* Suppose that *j* is a node in *T_i_* that is not relevant. In particular, *j* is not the root of *T_i_*. Let $j ^{\prime}$ be the LCA of the candidate sequences in ${M_{i , j}}$. Clearly, $j ^{\prime}$ is relevant and it is a strict descendant of *j*, and therefore, since ${T_{i , j ^{\prime} }}$ is a strict subtree of ${T_{i , j}}$, $\vert {M_{i , j}} \vert = \vert {M_{i , j ^{\prime} }} \vert$ while $\vert {N_{i , j}} \vert > \vert {N_{i , j ^{\prime} }} \vert$.    ■

Let $TP = \vert {M_{i , j}} \vert$, $FP = \vert {N_{i , j}} \vert$, $FN = \vert {M_i} \vert - \vert {M_{i , j}} \vert$, $TN = \vert {N_i} \vert - \vert {N_{i , j}} \vert$ and, similarly, let $TP ^{\prime} = \vert {M_{i , j ^{\prime} }} \vert$, $FP ^{\prime} = \vert {N_{i , j ^{\prime} }} \vert$, $FN ^{\prime} = \vert {M_i} \vert - \vert {M_{i , j ^{\prime} }} \vert$, $TN ^{\prime} = \vert {N_i} \vert - \vert {N_{i , j ^{\prime} }} \vert$. We have that $TP ^{\prime} = TP$, $FP ^{\prime} \le FP$, $FN ^{\prime} = FN$, $TN ^{\prime} \ge TN$, and $TN ^{\prime} + FP ^{\prime} = TN + FP$.

• Yule $\phi$: It has to be proved that
  \begin{align*}
 { \frac { TP ^ { \prime } \;TN ^ { \prime } - FP ^ { \prime } \;FN ^ { \prime } }  { \sqrt { ( TP ^ { \prime } + FP ^ { \prime } ) ( TP ^ { \prime } + FN ^ { \prime } ) ( TN ^ { \prime } + FP ^ { \prime } ) ( TN ^ { \prime } + FN ^ { \prime } ) } } } \ge { \frac { TP \;TN - FP \;FN }  { \sqrt { ( TP + FP ) ( TP + FN ) ( TN + FP ) ( TN + FN ) } } } 
\end{align*}
  Since $TN ^{\prime} + FP ^{\prime} = TN + FP$, $TP ^{\prime} + FN ^{\prime} = TP + FN$, $TP ^{\prime} = TP$, and $FN ^{\prime} = FN$, it suffices to prove that
  \begin{align*}
 { \frac { TP \;TN ^ { \prime } - FP ^ { \prime } \;FN }  { \sqrt { ( TN ^ { \prime } + FN ) ( TP ^ { \prime } + FP ^ { \prime } ) } } } > { \frac { TP \;TN - FP \;FN }  { \sqrt { ( TN + FN ) ( TP + FP ) } } } \tag { 1 } 
\end{align*}
  where $$TN ^{\prime} > TN$$ and $$FP ^{\prime} < FP$$.

We shall rewrite the numerators. It is straightforward to check that if we denote $TP + FN = {P_0}$, $FP + TN = FP ^{\prime} + TN ^{\prime} = {N_0}$, ${P_0} + {N_0} = M$, $TP + FP = P$, and $TP + FP ^{\prime} = P ^{\prime}$, then

\begin{align*}
 { \frac { TP \;TN ^ { \prime } - FP ^ { \prime } \;FN }  { \sqrt { ( TN ^ { \prime } + FN ) ( TP ^ { \prime } + FP ^ { \prime } ) } } } = { \frac { M \;TP - { P_0 } { \kern 1pt } P ^ { \prime } }  { \sqrt { P ^ { \prime } ( M - P ^ { \prime } ) } } } { \frac { TP \;TN - FP \;FN }  { \sqrt { ( TN + FN ) ( TP + FP ) } } } = { \frac { M \;TP - { P_0 } \;P }  { \sqrt { P ( M - P ) } } } 
\end{align*}

and, therefore, Equation (1) becomes

\begin{align*}
 { \frac { M \cdot TP - { P_0 } { \kern 1pt } P }  { \sqrt { P ( M - P ) } } } < { \frac { M \cdot TP - { P_0 } { \kern 1pt } P ^ { \prime } }  { \sqrt { P ^ { \prime } ( M - P ^ { \prime } ) } } } \tag { 2 } 
\end{align*}

where $0 < TP < P ^{\prime} < P < M$. Moreover, notice that $TP < {P_0}$ because *j* is not the root of *T_i_*.

Consider the function

\begin{align*}
\varphi ( x ) = { \frac { M \cdot TP - { P_0 } x }  { \sqrt { x ( M - x ) } } } 
\end{align*}

Equation (2) says that $\varphi ( P ) < \varphi ( P ^{\prime} )$ if $0 < P ^{\prime} < P < M$. So, to complete the proof of the statement, it is enough to prove that the function $\varphi ( x )$ is decreasing on $0 < x < M$. Its first derivative is

\begin{align*}
\varphi ^ { \prime } ( x ) = { \frac { M ( 2TP - { P_0 } ) x - { M^2 } \cdot TP }  { \sqrt { { { ( x ( M - x ) ) } ^3 } } } } 
\end{align*}

Then

\begin{align*}
\varphi ^{\prime} ( x ) < 0 \leftrightarrow ( 2TP - {P_0} ) x - M \cdot TP < 0
\end{align*}

Now, if $2TP \le {P_0}$, then $\varphi ^{\prime} ( x ) < 0$ for every *x*, while if $2TP > {P_0}$, then

\begin{align*}
\varphi ^ { \prime } ( x ) < 0 \leftrightarrow x < { \frac { M \cdot TP }  { 2TP - { P_0 } } } 
\end{align*}

and in this case

\begin{align*}
M < { \frac { M \cdot TP }  { 2TP - { P_0 } } } 
\end{align*}

because

\begin{align*}
M < { \frac { M \cdot TP }  { 2TP - { P_0 } } } \leftrightarrow 2M \cdot TP - M { P_0 } < M \cdot TP \leftrightarrow M ( TP - { P_0 } ) < 0
\end{align*}

and the latter inequality holds because $TP < {P_0}$. This implies that, also in this case, if $x < M$, then $\varphi ^{\prime} ( x ) < 0$.

• Area under the ROC curve: It has to be proved that
  \begin{align*}
 { \frac { TP ^ { \prime } ( FP ^ { \prime } + TN ^ { \prime } ) + TN ^ { \prime } ( TP ^ { \prime } + FN ^ { \prime } ) }  { ( TP ^ { \prime } + FN ^ { \prime } ) ( FP ^ { \prime } + TN ^ { \prime } ) } } \ge { \frac { TP ( FP + TN ) + TN ( TP + FN ) }  { ( TP + FN ) ( FP + TN ) } } 
\end{align*}
  We have that $( TP ^{\prime} + FN ^{\prime} ) ( FP ^{\prime} + TN ^{\prime} ) = ( TP + FN ) ( FP + TN )$ and $TP ^{\prime} ( FP ^{\prime} + TN ^{\prime} ) = TP ( FP + TN )$. Then, it suffices to prove that $TN ^{\prime} ( TP ^{\prime} + FN ^{\prime} ) \ge TN ( TP + FN )$. However, $TP ^{\prime} = TP$, $FN ^{\prime} = FN$, $TN ^{\prime} \ge TN$ and thus, the inequality follows.
• Rand index: It has to be proved that
  \begin{align*}
 { \frac { TP ^ { \prime } + TN ^ { \prime } }  { TP ^ { \prime } + FP ^ { \prime } + TN ^ { \prime } + FN ^ { \prime } } } \ge { \frac { TP + TN }  { TP + FP + TN + FN } } 
\end{align*}
  We have that $TP ^{\prime} = TP$, $FN ^{\prime} = FN$, $TN ^{\prime} \ge TN$, $FP ^{\prime} + TN ^{\prime} = FP + TN$ and thus, the inequality follows.

**Corollary 1.** *The Yule* ${Y_{i , j}}$*, the Youden* ${J_{i , j}}$*, the area under the ROC curve* ${A_{i , j}}$*, the Jaccard correlation coefficient* ${C_{i , j}}$*, and the Rand index* ${R_{i , j}}$ *only need to be computed for nodes j in T_i_ that are relevant.*

## 3. A Set Cover Approach to Taxonomic Annotation

Let us recall from Garey and Johnson (1979) that an instance of the set cover problem is a collection *C* of subsets of a finite set *X* whose union is *X*, and a solution to the set cover problem is a smallest subset $C ^{\prime} \subseteq C$ such that every element in *X* belongs to at least one member of $C ^{\prime}$. The set cover problem is non-deterministic polynomial time complete (NP-complete), but a logarithmic approximation can be computed in linear time (Johnson, 1974; Bar-Yehuda and Even, 1981) and an exact solution can be obtained by integer linear programming.

Recall also that in a metagenomic classification problem, there are often multiple candidate nodes in a reference taxonomy with the least classification error for a given read. As a set cover problem, the set of elements *X* is the set of candidate nodes in a reference taxonomy with the least classification error for the reads in a metagenomic sample, and the collection *C* of subsets of *X* is the collection of sets of candidate nodes in the reference taxonomy with the least classification error for each read.

**FIG. 2.**
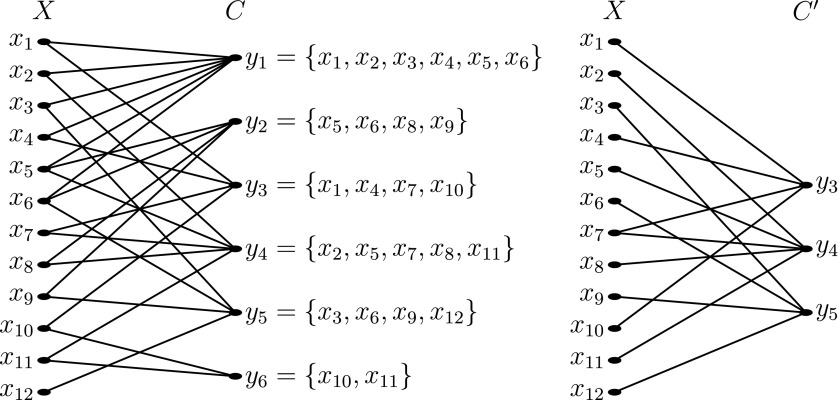
Left: A metagenomic classification problem viewed as a set cover problem. *X* is the set of reads from a metagenomic sample, and *C* is the collection of candidate nodes in the reference taxonomy with the least classification error for some reads from the metagenomic sample. Right: The smallest solution to the set cover problem instance.

The following example is adapted from Cormen et al. (2009, §35.3); see Figure 2.

**Example 1.** *Consider a metagenomic sample with reads* ${x_1} , \ldots , {x_{12}}$ *and candidate nodes in a reference taxonomy with the least classification error as follows:* $\{  {y_1} , {y_3} \}$ *for x_1_,* $\{  {y_1} , {y_4} \}$ *for x_2_,* $\{  {y_1} , {y_5} \}$ *for x_3_,* $\{  {y_1} , {y_3} \}$ *for x_4_,* $\{  {y_1} , {y_2} , {y_4} \}$ *for x_5_,* $\{  {y_1} , {y_2} , {y_5} \}$ *for x_6_,* $\{  {y_3} , {y_4} \}$ *for x_7_,* $\{  {y_2} , {y_4} \}$ *for x_8_,* $\{  {y_2} , {y_5} \}$ *for x_9_,* $\{  {y_3} , {y_6} \}$ *for* ${x_{10}}$*,* $\{  {y_4} , {y_6} \}$ *for* ${x_{11}}$*, and* $\{  {y_5} \}$ *for* ${x_{12}}.$ *Then, as an instance of the set cover problem,* $X = \{  {x_1} , \ldots , {x_{12}} \}$ *and* $C = \{  {y_1} \ldots , {y_6} \}$*, where* ${y_1} = \{  {x_1} , {x_2} , {x_3} , {x_4} , {x_5} , {x_6} \}$*,* ${y_2} = \{  {x_5} , {x_6} , {x_8} , {x_9} \}$*,* ${y_3} = \{  {x_1} , {x_4} , {x_7} , {x_{10}} \}$*,* ${y_4} = \{  {x_2} , {x_5} , {x_7} , {x_8} , {x_{11}} \}$*,* ${y_5} = \{  {x_3} , {x_6} , {x_9} , {x_{12}} \}$*, and* ${y_6} = \{  {x_{10}} , {x_{11}} \}$.

In a solution $C ^{\prime}$ to a metagenomic classification problem viewed as a set cover problem $( X , C )$, each read in *X* is annotated to a node in $C ^{\prime} \subseteq C$. Such a taxonomic annotation is not necessarily unique, and there may still be ambiguities in the classification of the metagenomic sample. For the problem instance from Example 1, the smallest solution is $\{  {y_3} , {y_4} , {y_5} \}$, which implies the taxonomic annotation of reads *x_1_*, *x_4_*, and ${x_{10}}$ to node *y_3_*, reads *x_2_*, *x_5_*, *x_8_*, and ${x_{11}}$ to node *y_4_*, reads *x_3_*, *x_6_*, *x_9_*, and ${x_{12}}$ to node *y_5_*, and read *x_7_* to either node *y_3_* or node *y_4_* in the reference taxonomy. The greedy algorithm of Johnson (1974) yields the approximate solutions $\{  {y_1} , {y_4} , {y_5} , {y_3} \}$ and $\{  {y_1} , {y_4} , {y_5} , {y_6} \}$.

The taxonomic annotation of a metagenomic sample can thus be seen as the reduction, and ideally the removal, of ambiguity in the identification of the reads in the metagenomic sample, where a read is ambiguous if it is annotated to more than one node in a reference taxonomy. Viewing the metagenomic classification problem as a set cover problem, an element of *X* is ambiguous if it belongs to more than one subset of the collection $C ^{\prime} \subseteq C$. The subsets of a set cover overlap on ambiguous elements.

**Definition 4.** *Let X be a finite set and let C be a collection of subsets of X whose union is X. The overlap of a set cover* $C ^{\prime} \subseteq C$ *is the total size of the subsets minus the size of X.*

Let the size of a set cover be the number of subsets of *X* that it contains, and let the total size of a set cover be the total size of the subsets of *X* that it contains. This corresponds to set cover problems I and II in Johnson (1974). It turns out that a set cover of smallest size does not necessarily have the least overlap, while a set cover of smallest total size always has the least overlap.

**Proposition 1.** *A set cover with the least number of subsets does not necessarily have the least overlap.*

*Proof.* Let $X = \{  1 , \ldots , n \}$ and assume, without loss of generality, that $n = 2k$ for $k \ge 3$. Let *S* be the following collection of subsets of *X*:

\begin{align*}
\{  1 , 2 \}  , \{  3 , 4 \}  , \ldots , \{  n - 1 , n \}  , \{  1 , \ldots , n - 1 \}  , \{  2 , \ldots , n \} 
\end{align*}

The set cover $\{  1 , \ldots , n - 1 \}  , \{  2 , \ldots , n \}$ has size 2, which is the smallest possible for *S* and *X*, and overlap *n*. The set cover $\{  1 , \ldots , n - 1 \}  , \{  n - 1 , n \}$ also has size 2, but it has overlap 1. Same for the set cover $\{  1 , 2 \}  , \{  2 , \ldots , n \}$, and *S* and *X* have no other set cover of size 2. However, the set cover $\{  1 , 2 \}  , \{  3 , 4 \}  , \ldots , \{  n - 1 , n \}$ has size $n / 2$ and overlap 0, which is the least possible overlap.    ■

The following result follows directly from Definition 4.

**Corollary 2.** *A set cover with the least total size of subsets has the least overlap.*

Based on the solution of a set cover problem with the least total size of subsets, the abundance profile of a metagenomic sample is given by the proportion of reads mapped to each node in the set cover, adjusted by a uniform distribution of any still ambiguous reads among all the nodes in the set cover that they are mapped to.

**Example 2.** *The relative abundance profile of the solution to the set cover view of the metagenomic classification problem of Example 1 is as follows:*

• *y_3_ has a relative abundance of* $( 1 + 1 + 0.5 + 1 ) / 12 = 29.17 \%$
• *y_4_ has a relative abundance of* $( 1 + 1 + 0.5 + 1 + 1 ) / 12 = 37.50 \%$
• *y_5_ has a relative abundance of* $( 1 + 1 + 1 + 1 ) / 12 = 33.33 \%$

## 4. Experimental Results

We have implemented the set cover approach to taxonomic annotation in a next release of the TANGO software (Clemente et al., 2011; Alonso et al., 2013), which belongs in the BioMaS (Fosso et al., 2015) and MetaShot (Fosso et al., 2017) pipelines. The new implementation of TANGO consists of the following:

• a first Python script for extracting the candidates matches for each read from the BLAST output,
• a second Python script for taxonomic annotation using the NCBI Taxonomy (Federhen, 2012, 2015), based on the ETE Toolkit (Huerta-Cepas et al., 2016),
• a third Python script for taxonomic annotation using the Greengenes taxonomy (McDonald et al., 2012),
• fourth Python script for resolving any remaining ambiguities by finding an exact solution to a set cover problem with the least total size of subsets, based on Gurobi Optimizer (Gurobi Optimization, Inc., 2017), and
• a fifth Python script for obtaining the relative abundance profile of the metagenomic sample.

While the second and third scripts process the input metagenomic sample one-sequence-read-at-a-time, the fourth script processes the output of the second or third script for the whole set of reads.

When using BLAST to map the reads to target sequences in the chosen reference taxonomy, the candidate matches for a read are those with the same E-value as the top hit. Notice that TANGO can be used with any read mapping tool alternative to BLAST [see Li and Homer (2010); Schbath et al. (2012) for a survey] by adapting the first script to the output format of the particular tool. Notice also that TANGO can be used for the taxonomic annotation of both amplicon reads (Tringe and Hugenholtz, 2008), with an amplicon reference taxonomy such as RDP (Cole et al., 2014), Greengenes (McDonald et al., 2012), or SILVA (Quast et al., 2013), and shotgun reads (Metzker, 2010), with a whole-genome reference taxonomy such as the NCBI Reference Sequence database (O'Leary et al., 2016).

**FIG. 3.**
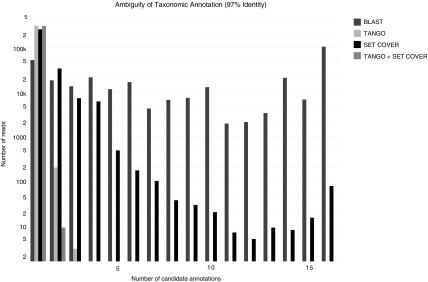
Histogram of BLAST matches, TANGO taxonomic annotations, nontaxonomic annotations with the set cover approach, and TANGO taxonomic annotations refined with the set cover approach, for the 302,581 reads from the human microbiome metagenomic data set and the 99,322 target sequences of the Greengenes taxonomy clustered at 97% identity. The rightmost bars correspond to 16 or more candidate annotations.

To assess the reduction in ambiguity of the set cover approach as opposed to the TANGO approach to taxonomic annotation, we have classified a representative subset of 302,581 reads from the human microbiome metagenomic data set of Caporaso et al. (2011) (available from ftp://ftp.microbio.me/qiime/tutorial_files/moving_pictures_tutorial-1.9.0.tgz) using the plain TANGO approach, the plain set cover approach, and the combined TANGO plus set cover approach. As illustrated in Figure 3, when mapping the 302,581 reads from the human microbiome metagenomic data set to the 99,322 microbial sequences in release 13.5 of the Greengenes taxonomy clustered at 97% identity and classifying them with TANGO, there are no reads with more than three candidate annotations and, when refining the TANGO output with the set cover approach, the number of unambiguous reads raises from 300,907 to 301,101, the number of reads with two candidate annotations drops from 200 to only 9, and the number of reads with three candidate annotations drops from 3 to 0.

Furthermore, to also assess the influence of the reference taxonomy in the taxonomic annotation of the metagenomic data set, we have mapped these 302,581 reads using release 2.2.31 of BLAST (Altschul et al., 1990) to the microbial sequences in release 13.5 of the Greengenes taxonomy (McDonald et al., 2012) clustered at various identity percent values, ranging from 61% to 100% (Table 4). The reduction in ambiguity follows a similar pattern: at 99% identity, the number of unambiguous reads raises from 300,916 to 301,111, the number of reads with two candidate annotations drops from 193 to only 1, and the number of reads with three candidate annotations drops from 3 to 0, and, at 100% identity, the number of unambiguous reads raises from 300,941 to 301,109, the number of reads with two candidate annotations drops from 171 to only 6, and the number of reads with three candidate annotations drops again from 3 to 0.

**Table 4.** Average Ambiguity of BLAST Matches, TANGO Taxonomic Annotations, Nontaxonomic Annotations with the Set Cover Approach, and TANGO Taxonomic Annotations Refined with the Set Cover Approach, for the 302,581 Reads from the Human Microbiome Metagenomic Data Set and the Greengenes Taxonomy Clustered at Various Identity Percent Values

		*Ambiguity*				
*Matches*	*BLAST*	*TANGO*	*Set cover*	*TANGO+*	*% Identity*	*Target sequences*
61	22	24,094	1.00012	1.00000	1.00012	1.00000
64	33	76,859	1.00007	1.00000	1.00007	1.00000
67	53	166,183	1.00028	1.00000	1.00028	1.00000
70	125	191,686	1.00388	1.00000	1.00388	1.00000
73	267	268,298	1.00806	1.00000	1.00803	1.00000
76	554	292,793	1.03580	1.00012	1.03575	1.00001
79	1165	297,070	1.04455	1.00015	1.04441	1.00000
82	2496	300,709	1.07380	1.00017	1.05724	1.00000
85	5088	300,998	1.08585	1.00057	1.07031	1.00000
88	10,544	301,067	1.21320	1.00038	1.07313	1.00000
91	22,090	301,096	1.76339	1.00026	1.15928	1.00000
94	46,256	301,109	3.91215	1.00082	1.34226	1.00000
97	99,322	301,110	16.97940	1.00068	1.24180	1.00003
99	203,452	301,112	64.43890	1.00066	1.40596	1.00000
100	1,262,986	301,115	161.81900	1.00059	1.47213	1.00002

The number of target sequences and the number of BLAST matches are also shown.

Finally, we have computed the relative abundance profiles at the phylum rank of the BLAST matches, the TANGO taxonomic annotations, the nontaxonomic annotations with the set cover approach, the TANGO taxonomic annotations refined with the set cover approach and, for reference, the QIIME taxonomy assignment using open-reference OTU picking (Navas-Molina et al., 2013; Rideout et al., 2014), for the 302,581 reads from the human microbiome metagenomic data set and the 99,322 microbial sequences of the Greengenes taxonomy clustered at 97% identity. As can be seen in Table 5, the four relative abundance profiles are consistent, with only minor differences between them and the QIIME relative abundance profile.

**Table 5.** Relative Abundance Profile of BLAST Matches, TANGO Taxonomic Annotations, Nontaxonomic Annotations with the Set Cover Approach, TANGO Taxonomic Annotations Refined with the Set Cover Approach, and QIIME (Open-Reference OTU Picking) for the 302,581 Reads from the Human Microbiome Metagenomic Data Set and the Greengenes Taxonomy Clustered at 97% Identity

*Taxonomic rank*	*BLAST*	*TANGO*	*Set cover*	*TANGO+*	*QIIME*
Archaea	0.013284	0.013284	0.013284	0.013284	0.015510
Crenarchaeota	0.013284	0.013284	0.013284	0.013284	0.015510
Bacteria	99.986716	99.986716	99.986716	99.986716	99.957297
Acidobacteria	0.074225	0.074391	0.074391	0.074391	0.036466
Actinobacteria	10.982357	10.982365	10.982365	10.982365	8.929160
Armatimonadetes	0.006642	0.006642	0.006642	0.006642	0.002070
Bacteroidetes	26.141444	26.141609	26.141443	26.141609	27.918210
Chloroflexi	0.091996	0.091993	0.091993	0.091993	0.018201
Cyanobacteria	2.564576	2.564843	2.564511	2.564843	1.989813
Deferribacteres	0.001328	0.001328	0.001328	0.001328	0.001742
Firmicutes	32.500312	32.463552	32.539437	32.463552	29.932524
Fusobacteria	3.802929	3.802929	3.802929	3.802929	4.529422
Gemmatimonadetes	0.029723	0.029889	0.029557	0.029889	0.001994
Planctomycetes	0.034207	0.034207	0.034207	0.034207	0.008272
Proteobacteria	21.029588	21.025871	21.028528	21.025871	25.774641
Spirochaetes	0.064096	0.064096	0.064096	0.064096	0.048609
Synergistetes	0.082141	0.119558	0.044834	0.119558	0.035557
Tenericutes	0.052810	0.052805	0.052805	0.052805	0.047571
Verrucomicrobia	2.395138	2.395138	2.395138	2.395138	0.601991
[Thermi]	0.085683	0.085683	0.085683	0.085683	0.054147
Other	0.047521	0.049817	0.046829	0.049817	0.026906
Unassigned	0.000000	0.000000	0.000000	0.000000	0.027193
Other	0.000000	0.000000	0.000000	0.000000	0.027193

All numbers are percentages.

## 5. Conclusion

We have addressed two potential sources of bias in the taxonomic annotation of metagenomic samples, which is usually done by first mapping the reads to the reference sequences and then classifying each read at a node in the clade of the LCA of the candidate sequences in the reference taxonomy with the least classification error. On the one hand, we have shown that the reference taxonomy being balanced or imbalanced does not affect the balance of the metagenomic classification problem, and we also shown that the Rand index is a better indicator of classification error for metagenomic classification problems than the often used area under the ROC curve and *F*-measure. On the other hand, we have reduced the taxonomic annotation problem for a whole metagenomic sample to a set cover problem, for which a logarithmic approximation can be obtained in linear time and an exact solution can be obtained by integer linear programming, and we have shown that a solution to the set cover problem with the least total size of subsets minimizes the ambiguity in the taxonomic annotation of the reads in a metagenomic sample.

We have also developed a proof-of-concept implementation of the set cover approach to taxonomic annotation in a next release of the TANGO software, as a series of Python scripts. Experimental results on a human microbiome metagenomic data set using BLAST and the latest release of the Greengenes taxonomy show that the set cover approach further reduces ambiguity in the taxonomic annotation obtained with TANGO without distorting the relative abundance profile of the metagenomic sample.

Future work includes extending the computation of balance ratio and total number of correct taxonomic annotations to the NCBI Taxonomy, taking ancestry relationships among the nodes in the reference taxonomy into account in the set cover formulation of the taxonomic annotation problem and last, but not least, extending the set cover problem formulation of the taxonomic annotation problem to a nontaxonomic metagenomic classification problem, with reference sequences but without a reference taxonomy.
